# Health and health behaviours before and during the Great Recession, overall and by socioeconomic status, using data from four repeated cross-sectional health surveys in Spain (2001–2012)

**DOI:** 10.1186/s12889-015-2204-5

**Published:** 2015-09-07

**Authors:** Xavier Bartoll, Veronica Toffolutti, Davide Malmusi, Laia Palència, Carme Borrell, Marc Suhrcke

**Affiliations:** Agència de Salut Pública de Barcelona, Barcelona, Spain; CIBER Epidemiología y Salud Pública (CIBERESP), Barcelona, Spain; Universitat Pompeu Fabra, Barcelona, Spain; Institute of Biomedical Research (IIB-Sant Pau), Barcelona, Spain; Health Economics Group, Norwich Medical School, University of East Anglia, Norwich, United Kingdom; Centre for Health Economics, University of York, York, United Kingdom; Centre for Diet and Activity Research (CEDAR), Institute of Public Health, Cambridge, United Kingdom

## Abstract

**Background:**

The objective of this study was to estimate changes over time in health status and selected health behaviours during the Great Recession, in the period 2011/12, in Spain, both overall, and according to socioeconomic position and gender.

**Methods:**

We applied a before-after estimation on data from four editions of the Spanish National Health Survey: 2001, 2003/04, 2006/07 and 2011/12. This involved applying linear probability regression models accounting for time-trends and with robust standard errors, using as outcomes self-reported health and health behaviours, and as the main explanatory variable a dummy “Great Recession” for the 2011/12 survey edition. All the computations were run separately by gender. The final sample consisted of 47,156 individuals aged between 25 and 64 years, economically active at the time of the interview. We also assessed the inequality of the effects across socio-economic groups.

**Results:**

The probability of good self-reported health increased for women (men) by 9.6 % (7.6 %) in 2011/12, compared to the long term trend. The changes are significant for all educational levels, except for the least educated. Some healthy behaviours also improved but results were rather variable. Adverse dietary changes did, however, occur among men (though not women) who were unemployed (e.g., the probability of declaring eating fruit daily changed by −12.1 %), and among both men (−21.8 %) and women with the lowest educational level (−15.1 %).

**Conclusions:**

Socioeconomic inequalities in health and health behaviour have intensified, in the period 2011/12, in at least some respects, especially regarding diet. While average self-reported health status and some health behaviours improved during the economic recession, in 2011/12, this improvement was unequal across different socioeconomic groups.

**Electronic supplementary material:**

The online version of this article (doi:10.1186/s12889-015-2204-5) contains supplementary material, which is available to authorized users.

## Background

After a period of economic growth since the beginning of 2000, the so-called ‘Great Recession’ hit the Spanish economy, provoking a tripling of the unemployment rate from 9.6 % in 2008 to 27.2 % in 2013 [1]. Over the same period real household incomes declined by 18.1 % on average, and the proportion of people at risk of poverty increased by 3.7 %, up to a level of 28.2 %, i.e., well above the European Union (EU-27) average (24.7 %) [2, 3]. Although this recession has been the most severe since the start of democracy in the late 1970s, there is currently little evidence about the extent to which this worsening of economic conditions may have affected the health and health behaviours of the Spanish population. The few studies that do exist suggest that while – for the population as the whole – mental health indicators appear to have worsened [4–7], mortality rates due to causes other than suicides have declined and self-reported general health has improved [8]. Yet, the average trends may obscure potentially heterogeneous effects between different socioeconomic groups, an aspect that has only been touched upon in the existing studies on Spain [5, 6].

Conceptual frameworks have highlighted the importance of the structural determinants, including social and economic policies and conditions that affect health behaviours and health outcomes [9]. The literature on the health effects of economic recessions reports mixed results for the population as a whole: while health behaviours and the majority of mortality indicators are not affected or improve (e.g., traffic accidents [10]); those of mental health or infectious diseases worsen [11]. Yet the population average results might well obscure important variations in the effects between different socioeconomic groups [12]. In Japan, for instance, self-reported health improved during the recession [13] and socioeconomic inequalities remained stable [14]. However, more stressful work environments may have adversely affected cause-specific mortality more among managers and professionals than among clerks and blue collar workers [15]. Empirical results regarding the health behaviour response to recessions have been more mixed: overall tobacco and alcohol consumption declined during a recession in Iceland [16, 17], in contrast to an increase in tobacco use among the unemployed in the UK [18] and in binge drinking in the UK and the USA [19, 20]. Obesity has been found to have decreased on average during recessions in the USA [10], but increased among lower socioeconomic groups in the USA and Italy [21, 22]. Other studies indicate that being at risk of unemployment reduces the consumption of fruits and vegetables and increases the consumption of unhealthy foods, including snacks and fast food [23].

The mechanisms proposed to explain the link between structural factors and individual health during economic recessions are complex and lead to ambiguous predictions about the expected health effects – a feature that may explain why effects can differ between socioeconomic groups. Three main potential mechanisms have been highlighted in the literature [24]: first, the reductions in working hours may induce either lower work-related stress, increase time for leisure, which may be devoted to health-promoting activities, such as physical exercise [25], more sleep, health care or an increase in the time spent cooking instead of consuming processed food. Counteracting these positive effects may be a lower quality of sleep, arguably in particular among the unemployed, as a result of their worsened mental health and well-being [26, 27]. Second, work-related stress may rise with the deterioration of working conditions, and workers may response by adopting unhealthy behaviours when not receiving the expected rewards, or may also act more cooperatively and adopt healthier behaviours in an effort to avoid dismissal [28]. Third, there could be an ‘income effect’, implying that with less disposable income the demand for normal goods tends to decrease and if these goods are harmful (beneficial) to health, people may obtain an improvement (worsening) in their health. Furthermore, it is noteworthy that in the context of austerity policies, such as those implemented in Spain, population health may be negatively affected by reduced investment in social and health-related public services, such as active labour market programs [29], infection control programs [30], or universal health care access [31].

The objective of this study was to estimate changes over time in health status and selected health behaviours during the Great Recession, in the period 2011/12, in Spain, both overall, and according to socioeconomic position and gender.

## Methods

Our sample was drawn from four waves (years 2001, 2003/04, 2006/07 and 2011/12) of the Spanish National Health Survey, a repeated cross-sectional study. The survey is nationally representative of the non-institutionalized Spanish population and covers – among the others – a considerable range of socio-economic and health related aspects. Ineligible cases (no data for 2001, 12.4 % for 2003/04, 10.3 % for 2006/07 and, 14.0 % for 2011/12, respectively) included vacant housing units, unavailable housing units, and housing units that were not residences. The response rates among eligible units in the last three waves were 77.0 %, 70.3 %, 71.8 % (no data for 2001), respectively. Ineligible sample units were replaced until the target number of interviews was achieved. No consent statement from participants was necessary as all microdata were anonymised and openly available online [32]. Our final sample consists of 47,156 individuals (9,252 for 2001, 10,840 for 2003/04, 15,470 for 2006/07 and 11,594 for 2011/12) aged between 25 and 64 years who were economically active (i.e., declaring to be either employed or actively seeking employment). To analyze the association between the Great Recession and health, we applied a Before-and-After design (where ‘before’ means ‘survey editions 2001 to 2006/07’ and ‘after’ means ‘the survey performed during the recession, in the period 2011/12’) [33], using the following model specification:

1 $$ {\mathrm{H}}_{\mathrm{i}}=\upalpha +\upbeta {\mathrm{X}}_{\mathrm{i}}+\updelta \mathrm{G}{\mathrm{R}}_{\mathrm{t}} + {\upeta}_{\mathrm{t}}+{\upvarepsilon}_{\mathrm{i}} $$

where H_i_ represents the health status or health behaviour indicator for individual i, X represents a set of individual characteristics (detailed below), *η*_*t*_ captures a linear trend and GR (Great Recession) represents a dummy variable which takes a value of 1 for the 2011/12 survey and 0 otherwise (i.e., for data from the 2001 to 2006/07 surveys). The trend *η*_*t*_ was introduced to allow for possible time-trends that need to be disentangled from the economic recession; ε is, to lead to unbiased estimation, assumed to be a mean zero error term, i) uncorrelated with the other observables (i.e., E(ε_i_ |GR_t_, η_t_, X_i_) = 0) and ii) uncorrelated with the treatment status conditional on the other covariates (i.e., E(ε_i_ |GR_t_, η_t,_ X_i_) = E(ε_i_ | η_t_, X_i_)). Assumptions i) and ii) are necessary conditions to obtain unbiased results. Our coefficient of interest is δ, which identifies the association between the Great Recession and health (or health behaviours). To estimate equation (1) we used a linear probability model, which according to Angrist [34] leads to closer results to those obtained in a binary model such as a logit or a probit. We used robust standard errors to control for potential autocorrelation in the error term. We also applied sampling weights in all the calculations in order to achieve population representativeness.

All the estimations are presented separately by gender. In order to assess whether and how the association varies by socioeconomic position, we stratify by employment status and educational level. We test whether the coefficients of each stratification are statistically different by means of F-test comparisons.

Our dependent variables included health status as well as a set of health behaviour proxies. Table 1 shows the definitions of the dependent variables used (see Additional file 1: Table A1 for a more detailed definition of the variables). Most of the health and health behaviour measures used are included in the recommendations of the European Health Interview Survey Guidelines (EHIS) [35] and are integrated in the Spanish health surveillance system indicators [36]. Self-reported general health has been shown to be a reliable measure of objective health [37], and a good predictor of mortality and other morbidities [38]. The obese and overweight categorization follows the Body Mass Index (BMI) guidelines for Spain [39] (see Additional file 1). Regarding the number of hours of sleep, we found no clear cut-off point for a categorization based on existing literature [40, 41], so we opted to use the continuous variable. Single-item questions on smoking, physical activity, and alcohol consumption serve to compute the prevalence of each variable. Self-reported smoking status has been shown to have a satisfactory correlation with more objective measures of smoking [42]. It has been suggested that measuring physical activity levels using single-item questions as employed here may be appropriate for establishing baseline data, and for differentiating between people who are sedentary and those who are vigorously active in large-scale samples [43, 44]. Self-reported alcohol consumption has demonstrated reasonable levels of reliability and validity [45], where a single-item question may capture population changes from moderate and light-drinkers to abstainers, with consequences for morbidity [46]. The cut-off point for heavy weekly alcohol consumption was calculated based on frequency, quantity and type of consumption, according to the World Health Organization (WHO) recommendations (see Additional file 1). The questions on the use of medicines and food consumption follow the EHIS recommendations. Food frequency questionnaires have been reported as a valid instrument for ranking subjects according to their level of intake, but not for estimating absolute intake [47], and have been found to be closely related to health outcomes [48].

**Table 1:** Definition of the dependent variables

Self-reported health	Binary variable equal to 1 if the respondent is in good or very good health.
Overweight /Obesity	Binary variable equal to 1 if the respondent is obese or overweight on the basis of body mass index.
Sleeping hours	Number of hours slept per day.
Smoking use	Binary variable equal to 1 if the respondent smokes.
Physical Activity	Binary variable equal to 1 if the respondent performs moderate or intense physical activity.
Alcohol last two weeks	Binary variable equal to 1 if the respondent drank any alcohol in the last two weeks.
Heavy drinking	Binary variable equal to 1 if the respondent drinks more than 17 Standard Basic Units of alcohol per week.
Tranquilizer or sleeping tablets intake	Binary variable equal to 1 if the respondent took at least one tablet in the last two weeks.
Vegetable consumption	Binary variable equal to 1 if the respondent eats vegetables daily.
Fruit consumption	Binary variable equal to 1 if the respondent eats fruit daily.
Legumes	Binary variable equal to 1 if the respondent eats legumes at least three times per week.
Fish consumption	Binary variable equal to 1 if the respondent eats fish at least three times per week.
Meat	Binary variable equal to 1 if the respondent eats meat at least three times per week.
Cold meat	Binary variable equal to 1 if the respondent eats processed meat (e.g., salami or sausages) at least three times per week.
Sweet food	Binary variable equal to 1 if the respondent eats sweet foods (e.g., jam, cookies, etc.) at least three times per week.

The main explanatory variable (GR) was a dummy variable equal to 1 during the Great Recession (the 2011/12 survey) and 0 otherwise (from the 2001 to 2006/07 surveys). The other controls we used (X_i_) included a list of socio-demographic variables: age; marital status (married as a reference category); regions of residence (17 autonomous communities, note that we dropped the autonomous cities of Ceuta and Melilla because of the small sample size); type of residential area coded in two main categories (rural area: municipality with ≤10,000 people, according to the Spanish census definition [49]; and urban area otherwise, serving as the reference category); employment status (using International Labour Organization (ILO) criteria, coded in two categories: employed as base category and unemployed); occupational social class (coded in five categories based on the current or last occupation of the respondent, or of the head of household for those never employed: high-level professionals and managers with more than 10 workers (i.e., the reference category) medium-level professionals and managers with less than 10 workers; intermediary and self-employed; supervisors and qualified or semi-qualified manual workers; and nonqualified workers) [50]; educational level (based on the International Standard Classification of Education (ISCED), as in [50]), defined in four categories: university degree (ISCED 8, 9, 10) as reference category, high secondary level (ISCED 5, 7), lower secondary or primary level (ISCED 3, 4, 6) and without any qualification or illiterate (ISCED 1, 2).

## Results

According to our descriptive statistics (see Additional file 1: Table A2), employment status was strongly affected by the Great Recession: the proportion of unemployment rose to 23.2 % for men and 22.8 % for women in 2011/12, compared to 7.9 % on average in 2007. However, the prevalence of good self-reported health decreased until 2006/07 but increased afterwards for both men and women.

### Health status

Tables 2 and 3 show associations between health or health behaviours and the Great Recession, in the period 2011/12, accounting for potential time trends and other covariates as expressed in equation (1) for men and women, respectively. In the first column we present the results for the overall sample, while in the remaining columns the results are stratified by employment status and education level.

**Table 2:** Marginal coefficients of the associations between the dummy variable ‘recession’ and health status, health behaviours and risk factors for men^a^

	Overall	Employment status			Education level				
		Employed	Unem-ployed	p^b^	University	High secondary	Lower secondary or primary	Without any qualification	p^c^
Men									
Health status									
Good self-reported health	0.076***	0.082***	0.076*	0.028	0.040*	0.079***	0.101***	−0.027	0.087
	(0.0118)	(0.0123)	(0.0427)		(0.0208)	(0.0213)	(0.0178)	(0.0833)	
Health behaviours and risk factors									
Overweight or obesity	0.012	0.016	0.011	0.824	−0.030	0.002	0.024	0.097	0.378
	(0.0145)	(0.0151)	(0.0497)		(0.0317)	(0.0295)	(0.0201)	(0.0904)	
Sleeping hours	0.090**	0.001	0.250*	0.058	0.061	0.183***	0.065	0.134	0.595
	(0.0353)	(0.0349)	(0.1495)		(0.0614)	(0.0682)	(0.0523)	(0.2535)	
Moderate or intense leisure physical activity^d^	0.032**	0.034**	0.014	0.874	0.019	0.068**	0.030*	−0.008	0.418
	(0.0134)	(0.0142)	(0.0449)		(0.0320)	(0.0289)	(0.0178)	(0.0389)	
Daily or occasional smokers	−0.008	−0.020	−0.022	0.147	−0.022	−0.016	0.005	−0.164**	0.161
	(0.0145)	(0.0153)	(0.0478)		(0.0294)	(0.0304)	(0.0206)	(0.0729)	
Alcohol last two weeks^d^	−0.054***	−0.051***	−0.022	0.257	−0.071***	−0.047*	−0.045**	−0.137*	0.623
	(0.0138)	(0.0144)	(0.0477)		(0.0273)	(0.0281)	(0.0195)	(0.0783)	
Heavy alcohol consumption^d, e^	0.020***	0.019***	0.015	0.529	0.002	0.008	0.031***	0.054*	0.012
	(0.0043)	(0.0044)	(0.0175)		(0.0083)	(0.0077)	(0.0062)	(0.0298)	
Tranquilizers or sleeping pills	−0.005	−0.006	−0.020	0.115	−0.008	−0.018*	−0.004	0.057	0.396
	(0.0057)	(0.0057)	(0.0241)		(0.0131)	(0.0103)	(0.0080)	(0.0483)	
Vegetables (daily)	−0.002	0.009	−0.063	0.004	−0.004	0.038	−0.013	−0.068	0.385
	(0.0141)	(0.0150)	(0.0448)		(0.0316)	(0.0289)	(0.0194)	(0.0734)	
Fruits (daily)	−0.091***	−0.074***	−0.121**	0.041	−0.045	−0.061**	−0.114***	−0.218***	0.060
	(0.0146)	(0.0154)	(0.0498)		(0.0319)	(0.0298)	(0.0204)	(0.0729)	
Legumes (3 times or more/ week)	0.034**	0.038***	−0.074	0.041	0.040	0.010	0.038**	−0.035	0.621
	(0.0135)	(0.0142)	(0.0479)		(0.0281)	(0.0280)	(0.0194)	(0.0681)	
Fish (3 times or more/ week)	−0.001	0.012	−0.068	0.055	−0.020	0.001	0.004	−0.003	0.937
	(0.0144)	(0.0152)	(0.0501)		(0.0319)	(0.0293)	(0.0200)	(0.0728)	
Meat (3 times or more/ week)	−0.097***	−0.093***	−0.110**	0.229	−0.093***	−0.079***	−0.092***	−0.268***	0.092
	(0.0125)	(0.0131)	(0.0457)		(0.0281)	(0.0251)	(0.0175)	(0.0705)	
Cold meat (3 times or more /week)	−0.047***	−0.047***	−0.003	0.341	−0.085***	−0.026	−0.036*	−0.173**	0.203
	(0.0148)	(0.0157)	(0.0496)		(0.0323)	(0.0304)	(0.0205)	(0.0806)	
Sweet food (3 times or more /week)	0.012	0.007	0.011	0.272	−0.017	0.025	0.031	−0.169**	0.067
	(0.0149)	(0.0158)	(0.0491)		(0.0325)	(0.0306)	(0.0208)	(0.0785)	

Robust standard errors in parentheses

Level of significance: * *p* < 0.10; ** *p* < 0.05; *** *p* < 0.01

^a^All models are adjusted by age, age^2^, marital status, region of residence (autonomous community), type of residential area (rural/urban), occupation and linear time trend. Spanish National Health Surveys 2001 to 2011/12

^b^Significance of *t*-test of the interaction between economic recession dummy and employment status

^c^Significance of likelihood ratio of the model with and without interaction between economic recession and education level

^d^Year 2003 not available

^e^Heavy alcohol consumption has been calculated only for weekly frequency consumption

**Table 3:** Marginal coefficients of the associations between the dummy variable ‘recession’ and health status, health behaviours and risk factors for women^a^

	Overall	Employment status			Education level				
		Employed	Unem-ployed	p^b^	University	High secondary	Lower secondary or primary	Without any qualification	p^c^
Women									
Health status									
Good self-reported health	0.096***	0.104***	0.100***	0.510	0.077***	0.083***	0.121***	0.135*	0.435
	(0.0131)	(0.0142)	(0.0342)		(0.0215)	(0.0268)	(0.0209)	(0.0812)	
Health behaviours and risk factors									
Overweight or obesity	−0.030**	−0.038**	−0.025	0.076	−0.028	−0.001	−0.067***	−0.186**	0.107
	(0.0146)	(0.0159)	(0.0358)		(0.0240)	(0.0297)	(0.0231)	(0.0875)	
Sleeping hours	0.153***	0.127***	0.161*	0.606	0.129**	0.192***	0.159***	0.187	0.933
	(0.0372)	(0.0397)	(0.0977)		(0.0631)	(0.0745)	(0.0590)	(0.2425)	
Moderate or intense leisure physical activity^d^	0.014	0.017*	0.011	0.402	0.065^***^	0.046^**^	−0.016	−0.007	0.014
	(0.0089)	(0.0099)	(0.0209)		(0.0205)	(0.0188)	(0.0115)	(0.0334)	
Daily or occasional smokers	0.044***	0.038**	0.050	0.578	0.019	0.007	0.063***	0.040	0.395
	(0.0140)	(0.0154)	(0.0348)		(0.0253)	(0.0299)	(0.0211)	(0.0715)	
Alcohol last two weeks^d^	−0.069***	−0.050***	−0.124***	0.054	−0.055*	−0.104***	−0.043*	−0.026	0.427
	(0.0151)	(0.0167)	(0.0356)		(0.0283)	(0.0312)	(0.0224)	(0.0783)	
Heavy alcohol consumption^d, e^	0.004	0.005	−0.001	0.998	−0.015	0.001	0.014***	−0.002	0.012
	(0.0040)	(0.0046)	(0.0089)		(0.0096)	(0.0086)	(0.0052)	(0.0021)	
Tranquilizers or sleeping pills	−0.024***	−0.037***	0.010	0.000	−0.026**	−0.012	−0.021	−0.183***	0.051
	(0.0084)	(0.0090)	(0.0215)		(0.0125)	(0.0170)	(0.0136)	(0.0596)	
Vegetables (daily)	−0.024	−0.017	−0.043	0.065	−0.013	0.034	−0.030	−0.271***	0.004
	(0.0150)	(0.0166)	(0.0364)		(0.0287)	(0.0313)	(0.0222)	(0.0795)	
Fruits (daily)	−0.079***	−0.071***	−0.106***	0.315	−0.058**	−0.048	−0.092***	−0.151*	0.429
	(0.0144)	(0.0159)	(0.0345)		(0.0268)	(0.0301)	(0.0216)	(0.0781)	
Legumes (3 times or more /week)	0.043***	0.039**	0.057*	0.392	0.051*	0.038	0.045**	−0.105	0.314
	(0.0136)	(0.0151)	(0.0325)		(0.0256)	(0.0274)	(0.0207)	(0.0805)	
Fish (3 times or more/ week)	−0.000	0.002	−0.007	0.437	0.042	−0.006	−0.013	−0.048	0.570
	(0.0149)	(0.0166)	(0.0354)		(0.0287)	(0.0307)	(0.0222)	(0.0840)	
Meat (3 times or more/ week)	−0.100***	−0.097***	−0.109***	0.567	−0.093***	−0.089***	−0.104***	−0.211***	0.541
	(0.0135)	(0.0149)	(0.0328)		(0.0258)	(0.0287)	(0.0197)	(0.0808)	
Cold meat (3 times or more /week)	−0.037**	−0.029*	−0.065*	0.836	−0.054*	−0.058*	−0.019	−0.099	0.558
	(0.0145)	(0.0161)	(0.0344)		(0.0276)	(0.0297)	(0.0218)	(0.0777)	
Sweet food (3 times or more /week)	0.001	0.002	−0.004	0.921	0.024	−0.011	0.001	−0.039	0.806
	(0.0152)	(0.0168)	(0.0361)		(0.0289)	(0.0312)	(0.0226)	(0.0848)	

Robust standard errors in parentheses

Level of significance: * *p* < 0.10; ** *p* < 0.05; ***p < 0.01

^a^All models are adjusted by age, age^2^, marital status, region of residence (autonomous community), type of residential area (rural/urban), occupation and linear time trend. Spanish National Health Surveys 2001 to 2011/12

^b^Significance of *t*-test of the interaction between economic recession dummy and employment status

^c^Significance of likelihood ratio of the model with and without interaction between economic recession and education level

^d^Year 2003 not available

^e^Alcohol consumption has been calculated only for weekly frequency consumption

Overall, self-reported health improved for both men and women. The probability of good self-reported health increased for men by 7.6 % in 2011/12, compared to the trend from 2001 until 2006/07, and for women by 9.6 %. Stratifying by employment status revealed similar increases but with employed men performing better than unemployed men. We found no differences by employment category for women. When stratifying the sample by educational attainment, there is a general improvement in self-reported health across all educational levels, except for men with the lowest educational attainment (incomplete primary education or illiterate), among whom there is no change, which implies a widening of inequalities by educational level.

### Health behaviours and risk factors

As for overweight and obesity, we found no significant change, during the Great Recession, in the period 2011/12, for men (overall and by educational level), but a reduction of 3.0 % in the prevalence for women. For women, the changes varied across educational levels, with a particularly large drop for those with lower educational levels.

The average number of hours of sleep per day has increased by about 0.9 h for men and by about 1.5 h for women. When splitting the sample by employment status the largest increase was among unemployed men (2.5 h), while there were no significant differences by employment status among women. With respect to education, we only found a significant difference among men with a high-school education, who had increased their hours of sleep by about 1.8 h, while for the other groups no significant changes were found. Among women, we found a general increase except for those without any qualification.

The prevalence of intense or moderate physical activity increased for men only, while for women there was no significant change, although the effect is heterogeneous among socio-economic groups. There was an increase among employed men (3.4 %), with a high school education (6.8 %), and a lower secondary or primary education (3.0 %), whereas no significant association was observed for women except for an increase among those who were employed (1.7 %) and/or highly educated (high secondary—4.6 %— or higher education — 6.5 %) representing a widening of educational level inequalities.

The prevalence of smoking increased among women (4.4 %), while there was no statistically significant association among men. Looking at specific groups, we found a decrease in the percentage of smokers among men without any qualification (16.4 %). Conversely, smoking increased for women in the lower secondary or primary education level (6.3 %).

The consumption of any alcohol has declined by 5.4 % for men and 6.9 % for women, which was consistent across all the groups except for unemployed men and for women without any qualification, for which there was no statistically significant change. By contrast, heavy alcohol consumption increased for men (2.0 %), the largest change being among those in the lowest education levels (i.e., by 3.1 % for those with a lower secondary or a primary education and by 5.4 % -significant at 10 % level only- for those without any qualification), increasing educational level inequalities. Among women heavy alcohol consumption significantly increased among those with a lower secondary or primary education (1.4 %).

There was no significant change in the intake of tranquilizers for men as a whole, but a slight decrease among those with high secondary education (1.8 %) and an increase among those without any qualification (5.7 %). Among women our results show a drop in intake of about 2.4 %, which was larger among those employed (3.7 %) and those without any qualification (18.3 %).

Tables 2 and 3 also show associations between the Great Recession, in the period 2011/12, and the frequency of consumption of selected food categories. Overall, no significant changes were found for the consumption of vegetables, fish and sweets, while there was a reduction in the consumption of fruits, meat, and cold meat, as well as an increase in the consumption of legumes. The average results mask considerable heterogeneity: unemployed men tended to decrease their fruit consumption by 12.1 %; looking at fruit intake by education level, the largest drop was among those in the lowest level (21.8 %). Among unemployed women the decrease in fruit consumption was 10.6 %. Stratifying by education, also the largest drop was among those in the lowest level (15.1 % significant at 10 % level only) widening educational level inequalities. With respect to the daily vegetable consumption, our results show a significant decrease (27.1 %) only for women without any qualification. The consumption of legumes increased for both men (3.4 %) and women (4.3 %). This increase is confirmed across employment status and educational levels, except for the insignificant results among men and women without any qualification and among unemployed men. Fish consumption was not significantly affected. The consumption of meat declined across all educational levels, with the largest drop among those in the lowest educated group, in both men and women. The consumption of cold meat decreased among men by about 5 %, with the largest decrease among those with the lowest education level (17.3 %), but no significant changes among unemployed men, while the employed dropped their cold meat consumption by 4.7 %. Similarly, for women the consumption of cold meat fell (3.7 %), with the largest decrease among unemployed women (6.5 %) as well as for those in the two groups with the highest educational attainment (5.4 and 5.8 %). The consumption of sweet foods remained unaffected, except for a sharp decrease among men without any qualification (16.9 %).

## Discussion

In Spain, while the first national health survey performed during the Great Recession, i.e., in 2011/12, appears to be associated with both an improvement in self-reported health and a range of health behaviours on average, these improvements hide important variations across educational levels, suggesting an increase in socioeconomic inequalities in self-reported health and in at least some health behaviours. For instance, men with the lowest educational level did not improve self-reported health, did not increase physical activity, and experienced a rise in the percentage of overweight/obesity as well as in their alcohol and tranquilizers consumption. Similarly for women, the adoption of healthier behaviours was concentrated among women with higher educational attainment.

### Health status

Self-reported health improved, in the period 2011/12, among men and women, both employed and unemployed, but to a lesser extent among the latter. Among those employed, beyond the potential improvement of health status induced by healthier behaviours, the ‘inhibitor effect’ to avoid dismissal might also have played a role. Despite the fact that working conditions worsened and job insecurity increased – as is usually observed during economic recessions [51, 52] – a further explanation might be that in a context of massive unemployment, being employed may lead one to overstate his or her own health perception compared with one’s unemployed peers. However, this compensation mechanism does not seem to occur among less educated men. The underlying idea could be that, in times of recession when unemployment rates rise, the pool of unemployed individuals tends to include a larger fraction of healthy people than otherwise, leading to an increase in aggregated self-reported health rather than a decrease. This is broadly in line with other studies suggesting that in times of high unemployment the association between unemployment and mortality weakens [53–55], and also that those engaged in low quality jobs may experience a short-term improvement in their self-reported health status when becoming unemployed [56]. Since unemployment benefits could be claimed for up to two years, overall self-reported health among the unemployed in 2011/12 may still not have worsened by as much as expected. Hence, the largest drop would be evident for those unemployed for more than two years. Unfortunately duration of unemployment was not available for all four surveys and we were unable to test its cumulative implications for health and health behaviours. A recent study found that in Sweden, men are levelling off in terms of self-perceived health but worsening their health-related behaviours with increased cumulative length of unemployment. Conversely, women’s health tended to deteriorate but no significant effects on their health-related behaviours have been found [57]. In Spain, the association between poor self-reported health and long-term unemployment was consistent between the survey editions of 2006/07 and 2011/12 (6).

While the Spanish government imposed a series of austerity measures during the recent recession - including cuts in public services, tightening of drug-prescription policies, tax increases and the closure of certain health care services, the downsizing of the public health system was not as severe as in Greece [30]. This difference may partially explain the variation in results between Spain and Greece in terms of changes in self-reported health, an overall decrease in self-reported health during the recession has been found for Greece [58], with some disadvantaged socio-economic groups suffering a particularly large deterioration [59]. From a policy perspective, this may underline the importance of maintaining some level of public goods and services provision even – and perhaps particularly so – in difficult economic times.

### Health behaviours and risk factors

In general we found that behaviours improved during the recession, i.e., in the period 2011/12, although our evidence suggests that the general improvement masks an exacerbation of some health behaviours during the recession in lower socio-economic groups. Our finding of no variation in smoking among men (but increased among women) should be taken with caution, because it might also be affected by the smoking bans introduced between 2005 and 2011. To examine this reasoning further, we compare our results with the national series of the number of cigarettes sold per capita per day for the period 1993–2009 [60], which shows a downward linear trend. We observed a decrease in alcohol consumption across educational levels, consistent with the income effect described in the introduction, except for unemployed men, where the income effect may have been counterbalanced by increased stress and by the selection mechanism, according to which – when unemployment rises – there is less selection in terms of health among employed and unemployed [61]. The increase in heavy weekly alcohol consumption for men and a moderate increase for women among the lowest educated group may derive from the increased mental health distress caused by the Great Recession especially among men [5, 7], as well as the observed clearly opposite gender patterns for tranquilizers intake, with men in the least educated group increasing their use while women’s tranquilizer intake remained unchanged.

Related to diet, on average we found a general decrease, in the period 2011/12, in the consumption of fruits, meat, and cold meat, an increase in that of legumes and no variation for the consumption of vegetables, fish and sweet foods. Hence it is impossible to judge the dietary changes of the recession as either unambiguously unhealthy or healthy. The picture is slightly clearer when looking at sub-groups: the Great Recession appears to have been somewhat detrimental for unemployed men, whose consumption of healthy foods such as fruit (legumes) decreased more (not increased) compared to those employed while not increasing legumes consumption as among the employed. Among women, we find employment status does not appear to affect changes during the recession in dietary proxies covered here.

In terms of protein sources, the results suggest that, in the period 2011/12, there was some substitution of legumes for meat as a source of cheap proteins, while our finding of no change for fish consumption may hide an increase in consumption of frozen fish at the expense of fresh fish. However, there is a very noteworthy differential association with the Great Recession, in the period 2011/12, across educational levels: the less educated group in particular has experienced a decrease in consumption that was almost double compared to the other groups, thus widening inequalities. The BMI-increase among less educated men, although not significant, might suggest an increase in fat intake. It is also worth noting that prolonged food-deprivation in the medium and/or long run may contribute to physical and mental health problems.

These findings support recent reports in the literature of limited or weak associations between the economic crisis and health behaviours for the overall population but with notable differences in the associations across sub-population groups [62, 63].

**Fig. 1:**
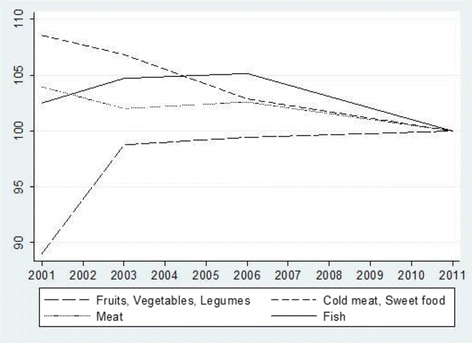
Average relative price ^a^of selected food categories, 2001-2011 (Base year 2011=100). ^a^Average relative price corresponds to the specific price by class or subclass of food category relative to the general price index for that year. Source: price indices were obtained from the Spanish National Statistical Office

Finally, we consider the contribution of prices and household incomes in shaping the associations between the Great Recession, in the period 2011/12, and health related behaviours, especially on food consumption, but also on smoking, alcohol and physical activity. Unfortunately, it was not feasible to add prices and incomes as independent variables to our baseline model, first because household incomes were missing for a large part of the sample across surveys (missing values ranging from 14.7 to 28.1 %): second, because there was a strong correlation among most prices and the trend variable; and third because our analysis of food consumption does not cover every single relevant food item or category but is based on a broad survey categorization that could hide substitution effects with other non included categories. However, we are in a position to develop some idea about the role of prices in changes in food consumption by looking at the evolution of relative prices from Fig. 1, and considering the price elasticity of food consumption previously estimated with household data^1^. Over the whole observation period the annual average relative price of fruits, vegetables and legumes increased by 12.4 % while prices for cold meat and sweet foods decreased by 7.9 %. From 2006 to 2011/12 the change in price level for fruits, vegetables and legumes was 0.0 %, −2.8 % for cold meat and sweet foods, −4.9 % for fish and −2.5 % for meat. Income and price elasticity for fish was lower than for meat, fruits and vegetables. With a drop in the prices of meat and fish and no variation in the prices of fruits, vegetables and legumes since 2006, the ‘income effect’ may have contributed significantly to the pattern of a reduction in consumption which we have found.

### Strengths and limitations

We have considered a wide range of health-related behaviours or risk factors, with data from four rounds of the National Health Survey in order to analyze how health and health-related behaviours have varied during the Great Recession, i.e., in the period 2011/12, in the short-term. Nevertheless, due to the lack of data for the first two years of the economic recession we cannot reject the hypothesis that health and health behaviours worsened during the first years and subsequently improved. The improvements we observe might reflect a mechanism of adjustment to the adverse situation. In order to be able to draw inferences about the long-term changes, more data would be required. In fact, since recovery in Spain is still sluggish, further (and possibly different) changes in health status and health behaviours may become evident in the longer term. However, since the unemployment rate soared very rapidly from 2008 through the first two years of the recession, we expect that the 2011/12 survey will already have captured much of the relevant health and health behaviour response. This kind of population survey may also incur bias in representativeness due to characteristics that are not controlled for in the sampling procedure, such as educational level and employment status. However, if such a bias exists, it should affect all survey rounds equally. We rely mostly on self-reported measures, which nevertheless have been validated and should reflect actual changes in the population.

Regarding our methodological approach, and as stressed in Meghir and Palme [33], the results of a Before-and-After estimation should be interpreted with caution, because it relies on the assumption that there was no other significant variation in the underlying macro-trends, except in our case for the Great Recession. While participation in training courses may be expected to increase during recessions, our descriptive data show a fairly constant trend for educational level in the overall adult population. This cohort effect has been captured by the linear trend. All the surveys achieved relatively high and reasonably similar response rates. We have also been limited by the use of repeated cross-sectional data in our analysis due to the availability of data, since we cannot observe the individual more than once, we cannot use panel techniques to purge the individual fixed effect. Although our study has investigated changes in population health status and health behaviours associated with the Great Recession, in the period 2011/12, we cannot establish whether this was a causal relationship.

Estimating the association between the Great Recession, in the period 2011/12, and health and health behaviours by using independent regressions on a set of common covariates could miss some factors that simultaneously affect the dependent variables through correlated error terms. For example, there could be a relation between overweight and smoking, or between tobacco and alcohol consumption, and among the kind of food eaten. To address these problems we repeated the analysis for the overall populations estimating seemingly unrelated equations (SURE) and the results (not reported, but available on request) showed no relevant difference in the strength and significance of the coefficients, hence indicating a low correlation of residuals.

## Conclusions

During the Great Recession, in the period 2011/12, in Spain, socioeconomic inequalities in health and health behaviours have intensified in at least some respects, especially for diet, which may also have adverse consequences on inequalities in health and mortality in the long-run. While average self-reported health status and some health behaviours improved during the economic recession, this improvement was heterogeneous across socioeconomic groups.

## Additional file

Additional file 1:
**Extended definition of variables and descriptive statistics.** (DOC 103 kb)

## Abbreviations

GR: Great Recession
EHIS: European health interview survey
BMI: Body mass index
WHO: World Health Organization
ILO: International labour organization
ISCED: International standard classification of education
SURE: Seemingly unrelated equations

## Footnotes

1 Income elasticity of 0.993 for fruits and vegetables, 1.052 for meat, 0.639 for fish; meaning for instance that a 10 % reduction of income will reduce expenditure of about 9.9 %, so fish appears to be more needed than the other. Uncompensated own price elasticity values are: −0.739 for fruits and vegetables, −0.656 for meat, and −0.184 for fish; meaning that a 10 % increase in fruit and vegetable prices reduces consumption about 7.4 % but only 1.84 for fish for the year 2003 from the Spanish Quarterly Household National Expenditure Survey 2003 [64].
