# Supplementary material for: Health and health behaviours before and during the Great Recession, overall and by socioeconomic status, using data from four repeated cross-sectional health surveys in Spain (2001–2012)
Source: BMC Public Health. 2015 Sep 7;15:865. doi: 10.1186/s12889-015-2204-5 (PMC4561448; doi:10.1186/s12889-015-2204-5)
Supplement: Additional file 1: — Extended definition of variables and descriptive statistics. (DOC 103 kb) [file 12889_2015_2204_MOESM1_ESM.doc]

**Table A1 Extended definition of variables.**

| Health status indicator: |
| --- |
| Self-reported health: interviewees were asked: “In the last twelve months, how would you describe your health status? very good, good, fair, poor or very poor?” Which was dichotomized with value 0 for fair, poor or very poor health and 1 for very good or good; |
| Health behaviours indicators and risks factors: |
| Overweight or obesity: body mass index (BMI) was computed from the self-reported height and weight following standard categorization^1^, with value 0 for normal and underweight and 1 for overweight or obesity. |
| Sleeping hours: interviewees were asked: “How many hours do you usually sleep?” We treat number of sleeping hours as a continuous variable. |
| Smoking: interviewees were asked: “Do you currently smoke?” Which was dichotomized with values 0 for never smoked or ex-smoker and 1 for occasional or daily smoker |
| Leisure-time physical activity: interviewees were asked: “How often do you practise physical activity in your leisure time?” Which was dichotomized with values 0 for those who reported to practise physical activity never or occasionally and 1 otherwise^2^. |
| Alcohol consumption: interviewees were asked: “Have you drunk alcohol in the last two weeks?” With answers taking values 0 for no consumption and 1 for any consumption^2^. |
| Heavy drinking: interviewees were asked about frequency of alcohol consumption: in surveys 2001 to 2006/07 response categories were weekly, monthly and less than monthly, while in 2011/12 they were asked about daily consumption in a specific week^2^. People drinking less than once a week were discarded. To calculate standard basic unit (SBU) of alcohol consumption we followed standard guidelines^3^. In line with the WHO [1], women drinking 17 SBU or more and men drinking 28 SBU or more per week were considered as heavy drinkers. To ease the interpretation we used a binary variable, which takes a value 1 for the heavy drinker and 0 otherwise. |
| Tranquilizer or sleeping tablets intake: with answers taking values 1 for those who took at least one tablet in the last two weeks and 0 otherwise. |
| Vegetables: with values 0 for less than daily and 1 for daily consumption^4^. |
| Fruits: with values 0 for less than daily and 1 for daily consumption. |
| Legumes: with values 0 for less than three times per week and 1 otherwise. |
| Fish: with values 0 for less than three times per week and 1 otherwise. |
| Meat: with values 0 for less than three times per week and 1 otherwise. |
| Cold meat: consumption of processed meat such as salami or sausages. With values equal to 0 for those who eat it less than three times per week and 1 otherwise. |
| Sweet food: consumption of foods such as jam, processed cookies etc. with values equal to 0 for those who eat them less than three times per week and 1 otherwise. |

^1^ BMI is a measure of body fat based on height and weight. Four categories are generally used to classify adults: (1) Underweight BMI≤ 18.5 kg/m^2^; (2) Normal weight BMI= 18.5–24.9 kg/m^2^; (3) Overweight BMI= 25–29.9; (4) Obesity BMI≥ 30 kg/m^2^.

^2^ Except for year 2003 which is not available in the survey. The reference period is not available except for 2006.

^3^ For wine, beer, aperitifs, and local beverages (such as cider) 10g of alcohol (1 SBU) were assigned for each intake, and for whisky, liqueurs and other combinations 20g (2 SBU).

^4^ For dietary habits from (7) to (14) we followed Spanish recommendations [2].

**References**

1. Ministerio de Sanidad y Consumo. Comisión clínica de la Delegación del gobierno para el plan nacional de drogas. Informe sobre el alcohol. Febrero 2007. Madrid. [Available at: <http://www.pnsd.msc.es/Categoria2/publica/pdf/InformeAlcohol.pdf> ]

2. Healthy food for all. Spanish Food Safety and Nutrition Agency (AESAN). Ministry of Health, Social Services and Equality: 2008. [Available at: <http://aesan.msssi.gob.es/AESAN/docs/docs/publicaciones_estudios/nutricion/informacion_nutricional_inmigrantes.pdf>]

Table A2 Descriptive statistics for active men and women 25-64 years. Spanish National Health Survey 2001-11/12.

|  | Men | | | | | Women | | | | |
| --- | --- | --- | --- | --- | --- | --- | --- | --- | --- | --- |
|  | 2001 | 2003/04 | 2006/07 | 2011/12 | p | 2001 | 2003/04 | 2006/07 | 2011/12 | p |
|  | N=5745 | N=6401 | N=8947 | N=6398 |  | N=3507 | N=4439 | N=6523 | N=5196 |  |
|  | % | % | % | % |  | % | % | % | % |  |
| Health status |  |  |  |  |  |  |  |  |  |  |
| Good self-reported health | 82.8 | 80.3 | 78.5 | 83.1 | 0.000 | 75.4 | 75.3 | 70.6 | 77.7 | 0.000 |
| Health behaviours |  |  |  |  |  |  |  |  |  |  |
| Overweight & obesity | 57.7 | 58.9 | 59.2 | 62.3 | 0.000 | 29.3 | 32.4 | 35.1 | 34.8 | 0.000 |
| Sleeping hours (average and t-value) | 7.22 | 7.28 | 7.19 | 7.27 | 0.016 | 7.27 | 7.21 | 7.17 | 7.24 | 0.000 |
| Moderate & intense physical activity | 18.6 | - | 25.9 | 31.7 | 0.000 | 11.8 | - | 15.0 | 18.6 | 0.000 |
| Daily and occasional smokers | 49.8 | 43.6 | 41.5 | 36.7 | 0.000 | 40.0 | 34.4 | 31.6 | 31.4 | 0.000 |
| Alcohol intake in the last 2 weeks | 75.3 | - | 74.5 | 69.0 | 0.000 | 48.0 | - | 49.5 | 44.4 | 0.000 |
| Heavy drinking per week | 2.3 | - | 1.3 | 2.2 | 0.002 | 1.1 | - | 0.8 | 0.9 | 0.122 |
| Tranquilizers last 2 weeks | 2.6 | 2.9 | 4.1 | 4.3 | 0.000 | 5.9 | 6.4 | 9.3 | 8.8 | 0.000 |
| Dietary behaviours |  |  |  |  |  |  |  |  |  |  |
| Vegetables (daily) | 25.4 | 37.0 | 33.8 | 41.1 | 0.000 | 38.1 | 46.3 | 47.8 | 53.8 | 0.000 |
| Fruits (daily) | 55.6 | 63.4 | 57.3 | 54.3 | 0.000 | 68.2 | 71.6 | 66.6 | 61.4 | 0.000 |
| Legumes (3 times or more/week) | 42.5 | 31.3 | 27.7 | 24.5 | 0.000 | 37.9 | 24.8 | 25.2 | 23.2 | 0.000 |
| Fish (3 times or more/week) | 42.8 | 43.5 | 36.0 | 35.5 | 0.000 | 49.8 | 46.1 | 41.4 | 40.3 | 0.000 |
| Meat (3 times or more/week) | 67.6 | 78.1 | 74.3 | 73.6 | 0.000 | 62.2 | 70.4 | 70.6 | 67.7 | 0.000 |
| Cold Meat (3 times or more/week) | 53.5 | 52.9 | 49.4 | 45.8 | 0.000 | 35.7 | 36.9 | 35.3 | 33.3 | 0.018 |
| Sweet food (3 times or more/week) | 49.3 | 45.2 | 43.8 | 43.9 | 0.000 | 52.7 | 46.4 | 46.9 | 44.9 | 0.000 |
| Socioeconomic variables |  |  |  |  |  |  |  |  |  |  |
| Education level |  |  |  |  |  |  |  |  |  |  |
| University degree | 18.8 | 18.7 | 21.9 | 19.4 | 0.000 | 24.5 | 28.0 | 27.2 | 26.2 | 0.000 |
| High secondary | 23.1 | 20.8 | 25.5 | 22.5 |  | 22.3 | 19.8 | 25.9 | 24.4 |  |
| Lower secondary or primary complete | 53.8 | 55.7 | 47.8 | 54.6 |  | 48.9 | 47.1 | 42.6 | 46.8 |  |
| Without any qualification | 4.1 | 4.8 | 3.9 | 3.5 |  | 4.2 | 5.1 | 3.2 | 2.6 |  |
| Missing | 0.2 | 0.0 | 1.0 | 0.0 |  | 0.1 | 0.0 | 1.2 | 0.0 |  |
| Occupational status |  |  |  |  |  |  |  |  |  |  |
| High-level professionals & managers  with >10 workers | 15.0 | 9.2 | 11.0 | 11.6 | 0.000 | 14.4 | 10.0 | 9.5 | 10.8 | 0.000 |
| Medium-level professionals &  managers with <10 workers | 5.5 | 11.5 | 10.3 | 8.2 |  | 8.4 | 11.6 | 10.8 | 10.0 |  |
| Intermediary & self-employed | 19.5 | 22.8 | 22.9 | 17.9 |  | 20.4 | 27.8 | 24.8 | 25.8 |  |
| Supervisors, qualified & semi-qualified | 51.4 | 44.8 | 40.5 | 50.1 |  | 43.6 | 32.0 | 32.6 | 30.2 |  |
| Non qualified | 4.9 | 11.2 | 13.5 | 11.8 |  | 9.8 | 18.3 | 21.4 | 22.3 |  |
| Missing | 3.8 | 0.6 | 1.7 | 0.5 |  | 3.3 | 0.2 | 1.0 | 0.9 |  |
| Employment status |  |  |  |  |  |  |  |  |  |  |
| Employed | 91.3 | 91.5 | 90.9 | 76.6 | 0.000 | 81.0 | 83.4 | 84.5 | 77.0 | 0.000 |
| Unemployed | 8.6 | 8.5 | 8.6 | 23.2 |  | 18.8 | 16.6 | 15.1 | 22.8 |  |
| Missing | 0.1 | 0.0 | 0.5 | 0.2 |  | 0.3 | 0.0 | 0.4 | 0.2 |  |
| Rural residence | 25.5 | 21.5 | 20.8 | 21.0 | 0.000 | 22.5 | 21.3 | 19.3 | 17.9 | 0.000 |
| Marital status : married | 66.6 | 66.9 | 61.6 | 63.3 | 0.000 | 58.7 | 61.0 | 61.5 | 58.9 | 0.000 |
| Age (mean and t-value) | 40.6 | 41.0 | 40.9 | 42.2 | 0.000 | 38.9 | 39.4 | 39.9 | 41.3 | 0.000 |

p value means differences across years
